# Women's preference for cesarean delivery and differences between Taiwanese women undergoing different modes of delivery

**DOI:** 10.1186/1472-6963-10-138

**Published:** 2010-05-26

**Authors:** Kuei-Hui Chu, Chen-Jei Tai, Chun-Sen Hsu, Mei-Chiang Yeh, Li-Yin Chien

**Affiliations:** 1 Department of Nursing, Chang Gung Institute of Technology, Taoyuan, Taiwan; 2 Department of Traditional Chinese Medicine, Taipei Medical University Hospital and Department of Medicine, Taipei Medical University, Taipei, Taiwan; 3 Department of Obstetrics and Gynecology, Taipei Medical University-Wanfang Hospital and Department of Medicine, Taipei Medical University, Taipei, Taiwan; 4 Institute of Clinical and Community Health Nursing, National Yang-Ming University, Taipei, Taiwan

## Abstract

**Background:**

The rate of cesarean delivery was 35% in 2007 in Taiwan. It is unclear how many of the cesarean deliveries were without medical indications. Women's preference for cesarean delivery during their course of pregnancy has rarely been studied and therefore our objectives were to examine rate of cesarean deliveries without medical indications, to explore women's preference for cesarean delivery as their gestation advances, and to compare background and perinatal factors among women who underwent different modes of delivery in Taiwan.

**Methods:**

This prospective study applied a longitudinal design. The study participants were 473 women who received prenatal care at four hospitals in Taipei and answered structured questionnaires at 20 to 24 weeks of pregnancy, 34 to 36 weeks of pregnancy, and 5 to 7 weeks after delivery.

**Results:**

Of the 151 women (31.9%) who had cesarean deliveries, 19.9% were without medical indication. Three indications: malpresentation, prior cesarean section, and dysfunctional labor together accounted for 82.6% of cesarean section with medical indications. The prevalence of maternal preference for cesarean delivery was found to be 12.5% and 17.5% during the second and third trimester, respectively. Of the women who preferred cesarean delivery during the second trimester, 93.2% eventually had a cesarean delivery. Women who were older, with older spouses, and who had health problems before or during pregnancy were more likely to have cesarean deliveries.

**Conclusions:**

About 20% of cesarean deliveries were without medical indications. Women's preference for cesarean delivery during the second trimester predicts subsequent cesarean delivery. Counseling regarding mode of delivery should be offered early in pregnancy, especially for women who are older or with older spouses, have health problems, or had a prior cesarean section.

## Background

Taiwan has a high rate of cesarean delivery. Chao reported that the rate of cesarean delivery in nine hospitals across Taiwan rose from 6.2 to 35 percent from 1950 to 1995 [1]. From 1998 to 2002, the cesarean section rate was about 34 percent [2]. Recent figures showed that the rate of cesarean section was between 32 and 34 percent during the period from 2002 to 2005 [3-8]. The rate of cesarean delivery was 35.15 percent in 2007 [9]. These rates are significantly higher than the 15 percent rate considered the highest acceptable cesarean delivery rate by the World Health Organization [10]. Cesarean deliveries increase both the health risks of mothers and infants and the costs of health care [11,12].

Taiwan, a modern Chinese country, has a National Health Insurance Scheme (NHIS) that provides coverage of general medical expenses to virtually all of its citizens, including indicated cesarean deliveries. Patients are free to choose any health care provider registered under the NHIS for their care, without the need for approval by a gatekeeper. Almost all health care providers have joined the NHIS. Despite the high rate of cesarean delivery in Taiwan, based on claim data from the NHIS, only 2.0 to 3.5 percent of cesarean births were upon mothers' requests for cesarean deliveries, without medical indications [13]. In order to decrease the high rate of cesarean delivery in Taiwan, there is a need for a more accurate estimate regarding the rate of elective cesarean deliveries. The Taiwan NHIS defines medical indications for cesarean deliveries as fetal distress, dysfunctional labor, antepartum hemorrhage, malpresentation, cord prolapse, induction failure, genital herpes, previous cesarean, prior uterine surgery, condyloma acuminata infections, treatable fetal congenital abnormalities, pre-eclampsia, infant weight <1500 g, abnormal pelvis, infant weight >4000 g, cephalo-pelvic disproportion, obstructive delivery, complications resulting from major internal diseases, and other special indications [14]. Medically indicated cesarean deliveries are fully covered by the NHIS, while cesarean deliveries without medical indication are paid for by women themselves. For cesarean delivery reimbursement, the NHIS reviews medical record case by case afterwards. If physician has recorded well-recognized indications for the cesarean delivery, it is highly likely that the health insurance payment will be passed. For our study, we defined cesarean delivery without medical indication by indications regulated by the NHIS. Though previous cesarean delivery is not a necessary medical indication for cesarean delivery, more than 95% of women opted for a cesarean section after a previous cesarean delivery in Taiwan [15]. Therefore, we treated previous cesarean section as a medical indication. We sought to identify cesarean deliveries with or without medical indications in order to shed light on unnecessary cesarean deliveries.

When women make the decision that they would prefer to have a cesarean delivery is important in determining the appropriate time to provide pregnant women with information regarding mode of delivery. However, women's preference for cesarean delivery during their course of pregnancy has rarely been studied. One study in Hong Kong examined the timing of the decision to have a cesarean delivery among women [16]. Their study found the prevalence of maternal preference for elective cesarean section was 17.2 percent in the second trimester and 12.7 percent in the third trimester. These authors further suggest that intervention programs should target the women at 20 weeks gestation who have a preference for elective cesarean delivery. The rate of cesarean deliveries was 10% higher in Taiwan than in Hong Kong. In addition, obstetric services are free in public hospitals, but there is a fee for service in private hospitals in Hong Kong, while in Taiwan, there are no differences in payment between the public and private hospitals. Therefore, the Hong Kong results cannot be generalized to the Taiwanese context. Previous studies of cesarean deliveries most often applied a cross-sectional study design and focused on medical factors. Maternal preference for cesarean delivery during their course of pregnancy is largely unexplored. In addition, previous studies have suggested that background and perinatal factors are associated with receiving a cesarean delivery, but few studies have distinguished cesarean deliveries with medical indications from those without medical indications. Our study objectives were to explore women's preference for cesarean delivery as gestation advances, to examine the rate of cesarean deliveries without medical indications, and to compare background and perinatal factors among women who underwent different modes of delivery (vaginal delivery, cesarean delivery with medical indications, and cesarean delivery without medical indications) in Taiwan.

## Methods

### Study participants

The study participants were recruited from two medical centers, a regional hospital, and a district hospital in the Taipei, Taiwan area. Pregnant women who received prenatal care at the four hospitals, who were over the age of 20 years, who had no pre-existing conditions or complications during early pregnancy such as high blood pressure, diabetes, hyperthyroidism, habitual abortion, who could read, write, and understand Mandarin Chinese, and whose pregnancy signs were stable were invited to participate in this study. We explained the study purpose and asked women to participate in this study when women applied for mother's manual at the hospitals (usually during first trimester). Those women who agreed to participate in the study signed a consent form and left their contact phone number and address for the research personnel. There were 682 women who met the inclusion criteria during the study period, a total of 574 (84.2 percent) women agreed to participate in this study. Of the 574 women, 527 (91.8 percent) completed the questionnaire at the second trimester, 486 (84.7 percent) completed it at the third trimester, and 473 (82.4 percent) women completed it one month after delivery. This study included the women who completed all the three questionnaires. We compared the characteristics of the women who completed all three questionnaires (n = 473) to those who completed the first questionnaire only (n = 54). There were no significant differences in mean age, marital status, educational level, or work status between the two groups.

### Data collection

A longitudinal questionnaire design was used for this study. The study participants filled out the structured questionnaires at 20 to 24 weeks of pregnancy, 34 to 36 weeks of pregnancy, and 5 to 7 weeks after delivery. Women who agreed to participate in this study received a phone call to inform them that they would receive the questionnaire by post. Questionnaires were mailed approximately 1 week before the indicated time. Women were asked to complete the questionnaires and return them using a pre-stamped, self-addressed envelope. A follow-up phone call was made if the questionnaire was not returned within 2 weeks. Data were collected from February 2006 through February 2007. This study was approved by the institutional review board at National Yang-Ming University.

### Measurements

A structured questionnaire was used to collect the data. The first questionnaire ([12-27] weeks of gestation) included background information (age, martial status, work status, educational level, and private health insurance), obstetrical history (parity, abortion, prior mode of delivery, disease history, planned pregnancy, gestational age, and health problems during pregnancy), and planned mode (34-36 weeks gestation) enquired about health problems during pregnancy and the planned mode of delivery. The third questionnaire ([6-8] weeks after delivery) enquired about the actual mode of delivery and reasons for the cesarean delivery if the women had a cesarean delivery. We classified the reasons for cesarean deliveries into the categories 'with' and 'without' medical indication using regulations by the NHIS. If the reasons were among the NHIS list of medical indications for a cesarean delivery, the cesarean delivery was classified as cesarean delivery with medical indication. If the reasons given for a cesarean delivery were not among those on the NHIS list, the delivery was classified as a cesarean delivery without medical indication.

### Sample size considerations

The rate of cesarean delivery in Taiwan was about 34% in year 2004. Therefore we assumed the two groups ratio (cesarean delivery vs. vaginal delivery) being 1:2. We assumed a group difference of medium effect size between the two groups (38% versus 25%, that is, 13% differences between cesarean and vaginal delivery groups). Based on the above, we would need about 450 participants for an α of 0.05 and a power of 0.80 using Sample Power version 2.0 for the calculation (SPSS Inc, Chicago, Illinois). If we assumed the follow-up rate to be 0.80, then 563 participants needed to be recruited at beginning of the study. Due to a lack of information in the previous literature, it was decided not to use the difference between cesarean delivery with and without medical indications in the sample size calculation.

### Data analysis

The data analysis was performed using the Statistical Package for the Social Sciences (SPSS) for Windows version 15.0 (SPSS Inc, Chicago, Illinois). Descriptive analyses performed including frequencies, percentages, means, and standard deviations. Group differences were examined using the chi-squared test.

### Result

The mean age of the study participants was 31.21 years (SD = 4.08, range: 16-43 years) and that of their spouses was 33.92 years (SD = 4.61, range: 18-52 years). About 59.4 percent (n = 281) of the women were primipara. Almost 11 percent (10.8 percent) of the women had a prior cesarean delivery. About 9 percent of women had a history of disease before this pregnancy, with 19 percent reporting health problems during the second trimester and 23 percent reporting health problems during the third trimester (Table 1).

**Table 1:** Characteristics of the study participants (n = 473)

Variable	All n (%)	Cesarean delivery with medical indications (n = 121) n (%)	Cesarean delivery without medical indications (n = 30) n (%)	Vaginal (n = 322) n (%)	P
Women's age, y					0.01
≤30	204 (43.1)	37 (30.6)	15 (50.0)	152 (47.2)	
31-40	263 (55.6)	81 (66.9)	14 (46.7)	168 (52.2)	
≥41	6 (1.3)	3 (2.5)	1 (3.3)	2 (0.6)	
Spouses' age					0.005
≤30	107 (22.6)	22 (18.2)	5(16.7)	80 (24.8)	
31-40	331 (70.0)	81(66.9)	23 (76.7)	227 (70.5)	
≥41	35 (7.4)	18(14.9)	2 (6.7)	15 (4.7)	
Disease before pregnancy					0.002
Yes	41 (8.7)	18 (14.9)	5 (16.7)	18 (5.6)	
None	432 (91.3)	103(85.1)	25 (83.3)	304 (94.4)	
Prior cesarean delivery					<0.001
Yes	51 (10.8)	51 (42.1)	0 (0.0)	0 (0)	
No	422 (89.2)	70 (57.9)	30 (100.0)	322 (100)	
Health problem during the second trimester					0.004
Yes	91 (19.2)	27 (22.3)	12 (40.0)	52 (16.1)	
No	382 (80.8)	94 (77.7)	18 (60.0)	270 (83.9)	
Health problem during the third trimester					0.009
Yes	110 (23.3)	35 (28.9)	12 (40.0)	63 (19.6)	
No	363 (76.7)	86 (71.1)	18 (60.0)	259 (80.4)	
Currently married					0.052
Yes	459 (97.0)	119 (98.3)	27 (90.0)	313 (97.2)	
No	14 (3.0)	2 (1.7)	3 (10.0)	9 (2.8)	
Women's work status					0.429
None	121 (25.6)	28 (23.1)	6 (20.0)	87 (27.0)	
Part time	30 (6.3)	8 (6.6)	0 (0.0)	22 (6.8)	
Full time	322 (68.1)	85 (70.2)	24 (80.0)	213 (66.2)	
Spouse work status					0.891
None	8 (1.7)	3 (2.5)	0 (0.0)	5 (1.5)	
Part time	37 (7.8)	9 (7.4)	2 (6.7)	26 (8.1)	
Full time	428 (90.5)	109 (90.1)	28(93.3)	291 (90.4)	
Women's educational level					0.593
High school or lower	83 (17.5)	23 (19.0)	2 (6.7)	58 (18.0)	
Vocational school	165 (34.9)	46 (38.0)	10 (33.3)	109 (33.8)	
University	176 (37.2)	41 (33.9)	15 (50.0)	120 (37.3)	
Postgraduate	49 (10.4)	11 (9.1)	3 (10.0)	35 (10.9)	
Spouse educational level					0.781
High school or lower	100 (21.2)	27 (22.3)	4 (13.3)	69 (21.4)	
Vocational school	126 (26.6)	30 (24.8)	7 (23.3)	89 (27.7)	
University	144 (30.4)	37 (30.6)	13 (43.3)	94 (29.2)	
Postgraduate	103 (21.8)	27 (22.3)	6 (20.0)	70 (21.7)	
Private health insurance					0.395
Yes	416 (87.9)	109 (90.1)	28 (93.3)	279 (86.6)	
No	57 (12.1)	12 (9.9)	2 (6.7)	43 (13.4)	
Parity					0.002
1	281 (59.4)	62 (51.2)	26 (86.7)	193 (59.9)	
>1	192 (40.6)	59 (48.8)	4 (13.3)	129 (40.1)	
History of abortion,					0.791
Yes	167 (35.3)	40 (33.1)	10 (33.3)	117 (36.3)	
No	306 (64.7)	81 (66.9)	20 (67.7)	205 (63.7)	
Planned pregnancy					0.646
Yes	288 (60.9)	78(64.5)	18 (60.0)	192 (59.6)	
No	185 (39.1)	43 (35.5)	12 (40.0)	130 (40.4)	
Birth gestational age					0.39
≤36 wks	18 (3.8)	3 (2.5)	1 (3.3)	14 (4.3)	
37-40 wks	409 (86.5)	111 (91.7)	25 (83.3)	273 (84.8)	
≥41 wks	46 (9.7)	7 (5.8)	4 (13.3)	35 (10.9)	

The overall rate of cesarean delivery among the study participants was 31.9 percent. Of the 151 women who had cesarean deliveries, 80.1 percent were medically indicated, while 19.9 percent were without medical indications. The major medical indications for cesarean deliveries were malpresentation (28.1 percent), prior cesarean delivery (28.1 percent), dysfunctional labor (26.4 percent), and fetal distress (8.3 percent) (Table 2).

**Table 2:** Reasons for a cesarean delivery (n = 151)

	n	%
Cesarean delivery with medical indications	121	80.1
Malpresentation	34	28.1
Previous cesarean	34	28.1
Dysfunctional labor	32	26.4
Fetal distress	10	8.3
Antepartum hemorrhage (including placenta previa)	4	3.3
Cephalo-pelvic disproportion	3	2.5
Multiple gestations	2	1.7
Pre-eclampsia	1	0.8
Infant weight >4000 grams	1	0.8
Cesarean delivery without medical indications	30	19.9

Women who had cesarean deliveries with medical indications (69.4% > 30 years of age) were older than women who had cesarean deliveries without medical indications (50%) and women who had vaginal deliveries (52.8%). Among women who had cesarean births without medical indications, spousal age was older (83.4 percent > 31 years), followed by women who had medically indicated cesarean deliveries (81.8 percent), and then by women who had vaginal deliveries (75.2 percent). Women who had cesarean deliveries were more likely to have had a history of disease before pregnancy, but among women with a cesarean delivery, the rate of having a history of disease was similar to that for women with and without medical indications for cesarean delivery. None of the women who had undergone a prior cesarean delivery eventually had a vaginal delivery. Health problems during the second and the third trimesters were more frequent among women who had cesarean deliveries without medical indications (both were 40 percent) than women who had cesarean deliveries with medical indication (22.3 and 28.9 percent) and women who had vaginal deliveries (16.1 and 19.6 percent). Women who had medically indicated cesarean deliveries were more likely to be multipara (48.8 percent) than women who had vaginal deliveries (40.1 percent) and women who had non-medically indicated cesarean deliveries (13.3 percent). Other factors (as listed in the Measurement section) were not significantly different among the three groups (Table 1).

The prevalence of maternal preference for cesarean delivery was 12.5% and 17.5% during the second and third trimester, respectively. Another 16 percent of women reported that they had not yet decided on a mode of delivery during the second trimester and 4 percent reported that they have not yet decided on a mode of delivery during the third trimester. Among women who had medically indicated cesarean deliveries, 37.2 percent planned for their eventual cesarean deliveries during their second trimester, while 52.9 percent planned for their eventual cesarean deliveries during their third trimester; another 25.6 percent of these women reported they had not yet decided on a delivery mode in their second trimester, while 5.0 percent reported they had not yet decided on a delivery mode during their third trimester. Among the women who had cesarean deliveries without medical indications, 33.3 percent planned for their eventual cesarean delivery during their second trimester, while 43.3 percent planned for their eventual cesarean delivery during their third trimester; another 13.3 percent of these women reported they had not yet decided on a delivery mode in their second trimester, while 10.0 percent reported they had not yet decided on a delivery mode during their third trimester (Table 3). There were no significant differences in the planned mode of delivery during pregnancy between women who received cesarean deliveries with and without medical indications (χ^2 ^= 3.22, df = 2, p = 0.20 for second trimester; χ^2 ^= 1.57, df = 2, p = 0.46 for t hird trimester).

**Table 3:** Planned mode of delivery during pregnancy and mode of delivery; n (%)

	Planned mode of delivery during the second trimester	Planned mode of delivery during the third trimester	Mode of delivery
All participants (n = 473)			
Cesarean	59 (12.5%)	83 (17.5%)	151 (31.9%)
Vaginal	338 (71.5%)	371 (78.4%)	322 (68.1%)
Undecided	76 (16.1%)	19 (4.0%)	
Cesarean with medical indications (n = 121)			
Cesarean	45 (37.2%)	64(52.9%)	
Vaginal	45 (37.2%)	51(42.1%)	
Undecided	31 (25.6%)	6 (5.0%)	
Cesarean without medical indications (n = 30)			
Cesarean	10 (33.3%)	13 (43.3%)	
Vaginal	16(53.3%)	14 (46.7%)	
Undecided	4 (13.3%)	3 (10.0%)	

**Figure 1:**
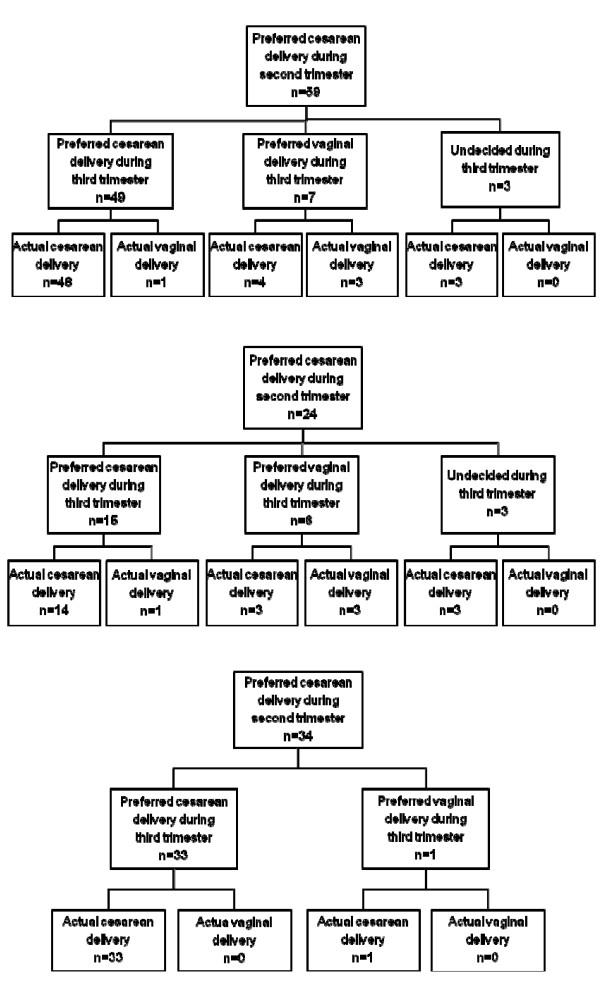
**Decision on mode of delivery among women who preferred cesarean delivery during second trimester (n = 59)**. Figure 1A. Decision on mode of delivery among primipara who preferred cesarean delivery during second trimester (n = 24). Figure 1B. Decision on mode of delivery among multipara with prior experiences of cesarean delivery who preferred cesarean delivery during second trimester (n = 34).

Of the women who received a cesarean delivery, 32.5% (59/151) and 51.0% (83/151) had preferred cesarean delivery during the second and third trimester, respectively (Table 2). To explore women's preference for cesarean delivery as gestation advances, we have presented the decision for mode of delivery among women who preferred cesarean delivery during the second trimester in Figure 1. Of the 59 women who preferred cesarean delivery during the second trimester, 49 women still preferred cesarean delivery during the third trimester; and 55 (93.2%) women had a cesarean delivery eventually. Of the 24 primipara who preferred cesarean delivery during the second trimester, 14 women still preferred cesarean delivery during the third trimester; and 20 (83.3%) women had a cesarean delivery eventually (Figure 1A). All of the three women who said they were undecided about mode of delivery during the third trimester had a cesarean delivery (Figure 1 and 1A). Only 1 out of the 140 multipara with prior experience of vaginal delivery preferred a cesarean delivery during the second trimester; and she eventually had a cesarean delivery. Of the 34 multipara with prior experience of cesarean delivery, 33 women still preferred cesarean delivery during the third trimester, and all of them had a cesarean delivery eventually (Figure 1B).

## Discussion

We found the rate of cesarean deliveries among the four participating hospitals was 31.9 percent, which was slightly lower than the Taiwanese national rate of 34.18 percent in 2006 and 35.15 percent in 2007 [15]. Given the context of high cesarean delivery rates, we found that 19.9 percent of cesarean deliveries were without medical indications. This rate underestimates the unnecessary cesarean deliveries in that not all women with prior cesarean deliveries must undergo cesarean deliveries, whereas in our study, women who had previous cesarean deliveries were classified as having medically indicated cesarean deliveries. Nonetheless, our cesarean delivery rate suggests that at least 19.9 percent of cesarean deliveries are unnecessary. Decreasing the number of cesarean deliveries without medical indications would decrease the overall rate of cesarean deliveries substantially in Taiwan (from 31.9 to 25.6 percent in this study). Contrary to our findings, studies in the US, UK, and Australia assert that maternal requests in the absence of clinical indications are not significant contributors to the cesarean section rate [17-19]. The differences could be attributed to differences in background cesarean rates, practice styles, and social-cultural contexts. Specifically, cesarean rates in Taiwan were substantially higher than in other countries. In addition, the system in Taiwan is loose in terms of regulating cesarean deliveries. If the woman or obstetrician chooses cesarean delivery without medical indications, it is not difficult for the obstetrician to find a medical explanation that will satisfy insurance guidelines since there is no gatekeeper system or pre-operation/concurrent review required for cesarean delivery under the Taiwan NHIS. Further study is needed to examine factors contributing to the differences across countries.

Previous reports using claim data from the Taiwan NHIS showed that 2.0 to 3.5 percent of cesarean births were upon the mothers' requests for cesarean deliveries without medical indications [13]. We found a much higher rate (19.9 percent) based on women's reports in our study. In the context that only cesarean deliveries with medical indications are reimbursed by the NHIS, the higher rate in our study suggests a more honest report than the claims data from the hospitals. A study using NHIS claims data in 2000 reported that the most common medical indications for cesarean section were prior cesarean section (44.1 percent), malpresentation (23.4 percent), and obstructive labor (15.6 percent) [15]. The most common medical indications in our study were malpresentation (28.1 percent), prior cesarean delivery (28.1 percent), and dysfunctional labor (26.4 percent). The reason for lower percentage of prior cesarean delivery in our study could be due to the decreased fertility rate in Taiwan in the past few years (60 percent of our study participants were primiparous). The previous study asserted that the proportion of fetuses with malpresentation delivered by cesarean section in Taiwan was twice the upper limit expected for all pregnancies, as indicated in international studies [15]. In their analysis, only 2 percent of cesarean deliveries were without medical indications, and they further suggested that physician claims could be dishonest to prevent nonpayment for cesarean deliveries in Taiwan [15]. Though the data were not directly comparable due to the large differences in women without medical indications, yet the three main indications were similar. Since malpresentation, dysfunction labor, and prior cesarean section accounted for 82.6 percent of cesarean deliveries with medical indications in our study, to decrease the overall cesarean delivery rate, more communication and educational programs should be conducted for both health care professionals and pregnant women regarding these three indications.

Our study found that most women had already decided on a mode of delivery in the second trimester. In addition, over 93% of women who preferred cesarean delivery during the second trimester had a cesarean delivery. It is usual in Taiwan that clinicians discuss the mode of delivery with women after 32 weeks of gestation. The results suggest that counseling about mode of delivery should be provided at an earlier time point. Most women did not change their decision regarding mode of delivery from the second to the third trimester, and a significant number of women decided their mode of delivery before the second trimester in our study. Identifying women who had a preference for cesarean delivery during the second trimester may increase the opportunity to influence their decision and thus reduce the rate of cesarean delivery. Mass media campaigns could be conducted to educate the general public about the risks associated with cesarean section and the benefits of vaginal birth in order to influence the decision earlier.

Women who were older, whose spouses were older, who had a history of disease before pregnancy, and who reported health problems during pregnancy were more likely to have cesarean deliveries. These factors were generally consistent with previous study findings [13,20-25]. We further found that women who underwent cesarean deliveries without medical indications were more likely to report health problems during pregnancy. Women who were older and who had health problems before or during pregnancy might fear for themselves or their babies to safely go through a vaginal delivery, coupled with the belief that a cesarean delivery is safest for the baby, therefore justifying the decision for a cesarean delivery [19]. Our results suggested that counseling regarding the method of delivery should be offered to women who report health problems before or during pregnancy and older women or spouses in order to address their related concerns.

All women with previous cesarean deliveries in our study underwent cesarean deliveries. A study showed that the rate of vaginal births after cesarean deliveries ranges from 3.9 to 4.5 percent in Taiwan [9]. In the US, the rate of vaginal birth after a cesarean section was 31 percent in 1998 and 12.7 percent in 2002 [26,27]. Concerns over uterine rupture and its attendant morbidity are associated with the decline in the trial of vaginal birth after cesarean delivery [28,29]. Nonetheless, the much lower rate of vaginal birth after cesarean birth in Taiwan suggests the need to counsel women who must choose between a vaginal birth trial and an elective repeated cesarean delivery after a prior cesarean delivery [30].

### Study limitations

For this study, data were collected by asking the pregnant woman herself, thus, clinical data and clinicians' perspectives are lacking. There may be concerns that women were not aware of the medical indications for their cesarean deliveries. However, we found that study participants described the reasons for their delivery method clearly, though we were unable to validate their answers.

While we divided cesarean deliveries into categories of 'with' and 'without' medical indications, we wish to be cautious and make it clear that a cesarean delivery without medical indications is not equivalent to a maternal request for cesarean deliveries. Since the decision as to the delivery mode is a negotiation process between the women, family, and clinicians, further study is needed to incorporate viewpoints from multiple sides in order to explore the decision process into more detail. Our sample size is limited for the group "cesarean delivery without medical indications," partly because we had no good estimates of the prevalence of cesarean delivery without medical indications from literature when we planned this study. Future studies can use our results to plan a more appropriate size for this group. This study sample is not a representative sample of the Taiwanese postpartum population. The rate of cesarean delivery in this study was slightly lower than the national rate, which partly could be due to the fact that this study excluded high-risk pregnancies. National data in Taiwan showed that in 2007 the mean age of women who delivered a baby was 29.8 years; that 52.8% of the mothers were primipara; and that 41.9% of the mothers had an educational level of university or higher [31]. When compared to our dataset, our study participants were older; had a higher educational level; and were more likely to be primipara. A population-based study is needed to examine this issue further.

## Conclusions

We found 19.9 percent of cesarean deliveries at the four participating institutions were without medical indications. Of the medically indicated cesarean deliveries, 82.6 percent were due to malpresentation, prior cesarean delivery, and dysfunctional labor. Women who preferred cesarean delivery during the second trimester, who were older, and who had health problems before or during pregnancy were more likely to have cesarean deliveries. Most women had already decided on a mode of delivery in the second trimester, which implied that health education programs regarding risks of cesarean delivery and benefits of vaginal delivery should be conducted among women and society at large. Counseling regarding mode of delivery could be offered earlier in pregnancy, especially for older women, women with older spouses, those with health problems, and those who have had a prior cesarean delivery. Identifying women who have a preference for cesarean delivery during the second trimester may increase the opportunity for intervention and thus reduce the rate of cesarean delivery. Nonetheless, more studies are needed to examine whether these strategies would decrease the overall rate of cesarean delivery.

## Competing interests

The authors declare that they have no competing interests.

## Authors' contributions

KC and LC participated in the design of the study, collected the data, performed the statistical analysis, drafted and revised the manuscript. CT gave advice in designing and interpreting the results, and co-wrote the manuscript. CH and MY gave advice in interpreting the results and revising the manuscript. LC secured funding for this study, supervised the whole study process, collected comments from all authors, revised and finalized the manuscript draft. All authors read and approved the final manuscript.

## Pre-publication history

The pre-publication history for this paper can be accessed here:

http://www.biomedcentral.com/1472-6963/10/138/prepub
